# A Patient Outcomes–Driven Feedback Platform for Emergency Medicine Clinicians: Human-Centered Design and Usability Evaluation of Linking Outcomes Of Patients (LOOP)

**DOI:** 10.2196/30130

**Published:** 2022-03-23

**Authors:** Alexandra T Strauss, Cameron Morgan, Christopher El Khuri, Becky Slogeris, Aria G Smith, Eili Klein, Matt Toerper, Anthony DeAngelo, Arnaud Debraine, Susan Peterson, Ayse P Gurses, Scott Levin, Jeremiah Hinson

**Affiliations:** 1 Department of Medicine Johns Hopkins University School of Medicine Baltimore, MD United States; 2 Center for Social Design Maryland Institute College of Art Baltimore, MD United States; 3 Department of Emergency Medicine Johns Hopkins University School of Medicine Baltimore, MD United States; 4 StoCastic Towson, MD United States; 5 Armstrong Institute Center for Health Care Human Factors Johns Hopkins Medicine Baltimore, MD United States; 6 Department of Anesthesiology and Critical Care Medicine Johns Hopkins University School of Medicine Baltimore, MD United States

**Keywords:** emergency medicine, usability, human-centered design, health informatics, feedback, practice-based learning and improvement, emergency room, ER, platform, outcomes, closed-loop learning

## Abstract

**Background:**

The availability of patient outcomes–based feedback is limited in episodic care environments such as the emergency department. Emergency medicine (EM) clinicians set care trajectories for a majority of hospitalized patients and provide definitive care to an even larger number of those discharged into the community. EM clinicians are often unaware of the short- and long-term health outcomes of patients and how their actions may have contributed. Despite large volumes of patients and data, outcomes-driven learning that targets individual clinician experiences is meager. Integrated electronic health record (EHR) systems provide opportunity, but they do not have readily available functionality intended for outcomes-based learning.

**Objective:**

This study sought to unlock insights from routinely collected EHR data through the development of an individualizable patient outcomes feedback platform for EM clinicians. Here, we describe the iterative development of this platform, Linking Outcomes Of Patients (LOOP), under a human-centered design framework, including structured feedback obtained from its use.

**Methods:**

This multimodal study consisting of human-centered design studios, surveys (24 physicians), interviews (11 physicians), and a LOOP application usability evaluation (12 EM physicians for ≥30 minutes each) was performed between August 2019 and February 2021. The study spanned 3 phases: (1) conceptual development under a human-centered design framework, (2) LOOP technical platform development, and (3) usability evaluation comparing pre- and post-LOOP feedback gathering practices in the EHR.

**Results:**

An initial human-centered design studio and EM clinician surveys revealed common themes of disconnect between EM clinicians and their patients after the encounter. Fundamental postencounter outcomes of death (15/24, 63% respondents identified as useful), escalation of care (20/24, 83%), and return to ED (16/24, 67%) were determined high yield for demonstrating proof-of-concept in our LOOP application. The studio aided the design and development of LOOP, which integrated physicians throughout the design and content iteration. A final LOOP prototype enabled usability evaluation and iterative refinement prior to launch. Usability evaluation compared to status quo (ie, pre-LOOP) feedback gathering practices demonstrated a shift across all outcomes from “not easy” to “very easy” to obtain and from “not confident” to “very confident” in estimating outcomes after using LOOP. On a scale from 0 (unlikely) to 10 (most likely), the users were very likely (9.5) to recommend LOOP to a colleague.

**Conclusions:**

This study demonstrates the potential for human-centered design of a patient outcomes–driven feedback platform for individual EM providers. We have outlined a framework for working alongside clinicians with a multidisciplined team to develop and test a tool that augments their clinical experience and enables closed-loop learning.

## Introduction

Proficiency in the practice of medicine is achieved over years of rigorous training and is maintained through a lifelong commitment to practice-based learning and improvement [1]. This is among the core competencies described by the Accreditation Council for Graduate Medical Education (ACGME) for all physician trainees and has been integrated into the Maintenance of Certification program by the American Board of Medical Specialties (ABMS) [2,3]. To engage in practice-based learning, clinicians must continuously assess the effectiveness of their own clinical practice [4] and actively work to make improvements at the individual and system levels [5]. Optimal experiential learning requires robust feedback mechanisms; therefore, the learner understands the real-world consequences of the actions taken and is provided the opportunity to correct course in response to suboptimal outcomes [6]. This type of closed-loop learning is a core component of deliberate practice and has been central to medical education since the time of William Osler [7]. However, the availability of outcomes-based feedback is highly variable across practice settings and medical specialties [8,9] and clinicians are often unaware of how their actions affect the short- and long-term health of patients [10].

Emergency medicine (EM) clinicians play a pivotal role in the health care system; yet, practice in an environment makes outcomes-driven learning particularly challenging. Emergency departments (EDs) are a point of entry for acutely injured and critically ill patients and are a primary source of health care for vulnerable populations [11]. In 2016, ED encounters exceeded 145 million in the United States alone [12]. While making high-stakes decisions under excessive cognitive loading and time pressure [12-15], EM clinicians set care trajectories for the majority of hospitalized patients and provide definitive care to an even larger number who are discharged into the community [16]. Additionally, because of the episodic nature of emergency care, longitudinal doctor-patient relationships do not exist in the ED. Currently, there is no mechanism for delivering systematic information about post-ED encounter patient outcomes to emergency clinicians for patient outcome–based feedback [10,17]. Emergency clinicians recognize the value of practice-based and outcomes-informed experiential learning, and they are interested in more robust postencounter feedback systems. These systems have shown potential to decrease adverse ED events, improve team function, and further clinician professional development [3,6,17,18]. Currently, postencounter telephone calls to patients of interest and case conferences (eg, morbidity, mortality) are the most common methods used to elicit postencounter patient outcome feedback in EM [17]. Over the past decade, the widespread adoption of electronic health records (EHRs) has generated continuously growing pools of clinical data, including data related to post-ED encounter patient outcomes with the potential to inform clinician practice and facilitate practice-based learning [19,20]. To date, this potential has not been realized.

In this study, we sought to unlock insights from routinely collected EHR data through the development of an individualizable patient outcomes–feedback platform. Here, we describe the iterative development of this platform, Linking Outcomes of Patients (LOOP), under a human-centered design (HCD) framework [21] and execute this through a unique collaboration between the Johns Hopkins Schools of Medicine and Engineering as well as Maryland Institute College of Art (MICA). We also report the functionality and usability of LOOP as assessed by the direct measurement of the end user clinicians’ knowledge, skills, and attitudes as they interacted with and used LOOP.

## Methods

### Research Team Structure and Study Population

This mixed methods study was performed between August 2019 and February 2021 via a collaborative effort between the Center for Social Design at MICA and the Center for Data Science in Emergency Medicine (CDEM) at the Johns Hopkins University School of Medicine in Baltimore, Maryland. Our core study team comprised EM physicians, design researchers, human factors engineers, software engineers, and data analysts. This project was conducted in 3 phases: (1) conceptual development under an HCD framework, (2) technical platform development, and (3) usability evaluation. Study sites included a large quaternary academic medical center ED and a community hospital ED; all study participants were EM clinicians who practiced at one of these sites.

### Ethics Approval

This study was approved by our institutional review board (IRB00185078) after expedited review.

### Phase 1: Conceptual Development Under an HCD Framework

**Figure 1:**
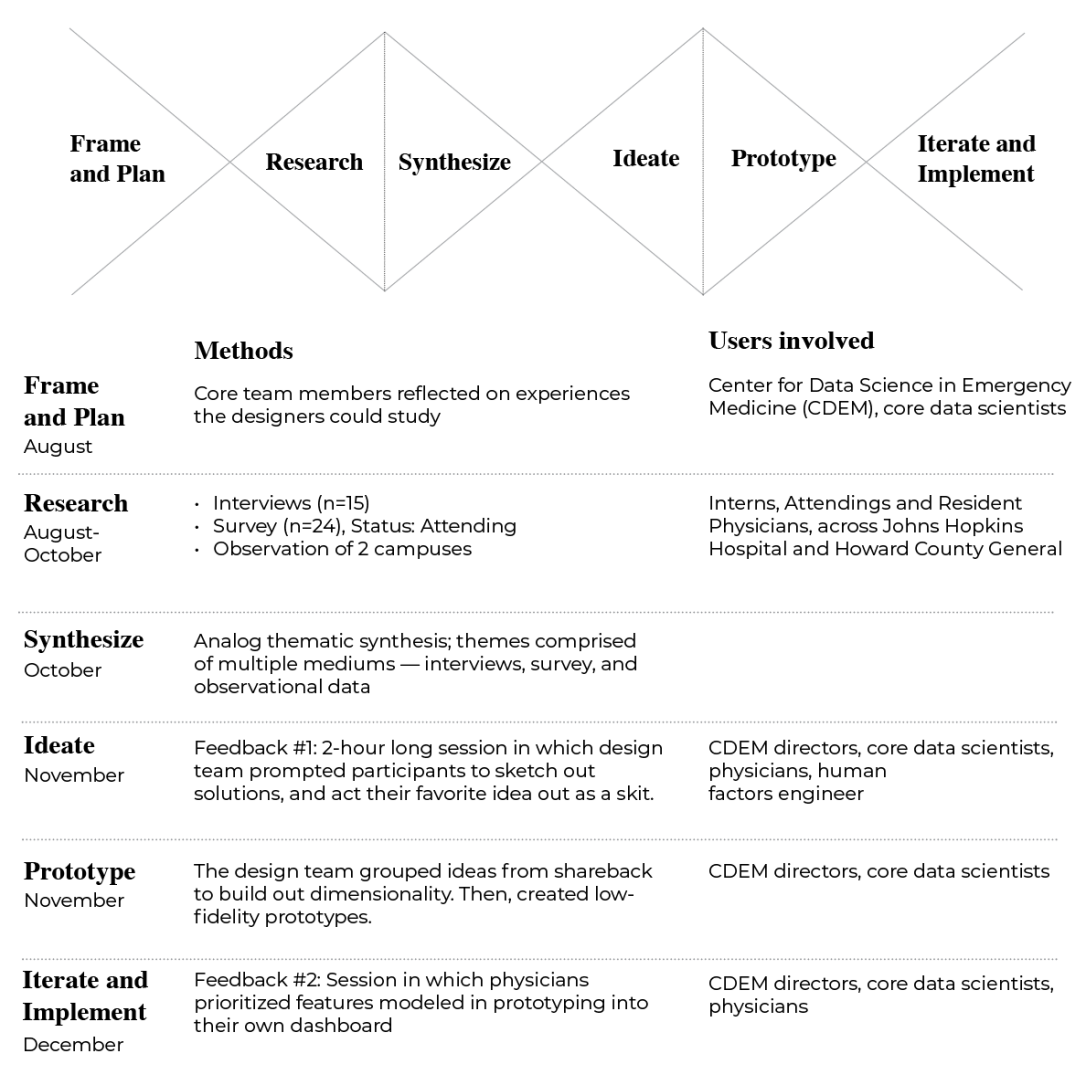
Human-centered design methods used to develop Linking Outcomes Of Patients. CDEM: Center for Data Science in Emergency Medicine.

In the fall of 2019, we conducted an intensive 16-week HCD studio [21] focused on addressing the delivery of feedback to EM clinicians related to post-ED encounter patient outcomes. MICA design faculty (BS and CM) led the studio in partnership with CDEM researchers. As shown in Figure 1, our HCD studio consisted of 6 stages: *frame and plan, research, synthesize, ideate, prototype,* and *iterate and implement.*

First, our multidisciplinary research team conducted cocreation sessions to fine-tune the scope and objectives of the project. We then engaged in design research with end users via a mixed methods approach that included observations, semistructured interviews, and surveys of EM clinicians. Observations focused on EM clinician interactions with existing technologies, and semistructured interviews focused on current patient outcome follow-up and practice-based learning behaviors (Table 1). Surveys were used to assess current patient follow-up practices, identify important patient outcomes for post-ED encounter follow-up, and define ideal timeframes for outcome reporting (Multimedia Appendix 1). Thematic analysis of research output was used to synthesize “personas,” “design principles,” and “opportunity areas” that would guide future HCD studio activities.

**Table 1:** Examples of high priority questions from the design research stage interview guide.

Question category	Examples
Rapport building	What does a perfect day at the emergency department look like for you?
Stress	What things currently complicate your decision making in the emergency department? (individual, institutional, environmental) *Optional follow up*: Could you relate that back to feedback?
Feedback	How do you find out what happens to your patients after they leave the emergency department? *Optional Follow up*: Why do you think you don’t receive the kind of information/feedback you would like?
Design	What format would prefer to receive feedback on what happens to respiratory infection patients and why? (Prompt: ask about how they prefer seeing info, ie, text, charts, images, video, voice messages)
Diagnostic/decision making	Walk me through how you develop a set of possible diagnoses and how you differentiate between them to come to a final diagnosis.

Next, end users were reincorporated into the HCD studio through an in-person multistakeholder ideation session that also included engineers and data analysts. Previously defined *design principles* and *opportunity areas* were employed as guides, and potential solutions were generated using numerous ideation techniques (eg, brainstorming, brain dumping, sketching, storyboarding). The ideation session output was subsequently used by designers to develop a series of solution prototypes. Prototypes progressed through several levels of fidelity as designers collaborated with clinical and technical research team members to ensure that solutions were compatible with existing health information technology. Finally, designers and end users convened to develop an idealized version of the feedback tool, which incorporated features generated during the ideation and prototyping stages. *User personas* were used as a touch point to build out dimensionality for the ideal feedback tool. Attendees of these sessions also created an *experience map*, which described the envisioned end user’s journey with the tool through 4 phases: learning about the tool, using the tool, owning the tool, and implementing the tool into practice.

### Phase 2: Technical Platform Development

#### Patient Outcome Selection

An initial set of patient outcomes was selected for inclusion in the initial version of our tool based on end user preferences (discovered through *design research)* and feasibility of data collection and standardization. Outcomes selected were in-hospital mortality, escalation of level of care (eg, floor to intensive care unit) within 24 hours of ED departure, and return to the ED within 72 hours of discharge. These outcomes are commonly used quality measures [22,23], applicable to the entire ED population, and reliably recorded in the EHR.

#### Data Capture, Normalization, and Delivery

CDEM data analysts and engineers developed a data processing pipeline to facilitate the population of post-ED encounter patient outcomes within our feedback platform. In brief, raw EHR data were first populated within a Health Insurance Portability and Accountability Act–compliant research computing environment, where a normalization code was developed to identify and label post-ED encounter patient-level events and to attribute patient encounters to individual EM clinicians by using native EHR data fields and timestamps. The normalization code was validated via chart review by an EM clinician and data analyst. Following validation, the normalization code was applied to daily extracts of EHR data within a reporting server, and aggregated views of normalized data were pushed to a presentation server that would power our patient feedback platform.

#### Digital Feedback Platform Design

Working alongside EM clinicians (JH, CK, and SP) and software engineers (AGS and MT) in a co-design process, our lead design researcher (CM) transitioned the final analog prototype into a fully operational digital platform. Early digital prototypes were built using dummy data and design software that facilitated realistic end-user interaction and rapid iterative improvement (Agile methodology) [24,25]. User needs, including minimum information requirements, optimal outcome definitions, and data labels and data filtering capacities, were further defined within this environment. A final digital mock-up was then used as a template to develop LOOP within the clinician-facing analytic software used by our institution to ensure that our final product adhered to the principles and requirements generated through co-design and would function within our local information technology infrastructure. Throughout this process, our design researcher also worked closely with data analysts and software engineers who led the data processing pipeline development to ensure interoperability of our entire LOOP system.

### Phase 3: Usability Evaluation

Usability evaluation was performed by 3 members of the research team: a frontline EM physician (CK), a design researcher (CM), and a health systems engineer with a clinical background (ATS). All participants included were practicing clinicians at an ED study site; members of our study team were excluded from participation in the usability evaluation. Participants were selected using a purposive stratified sample of EM clinicians with representation from multiple end user groups based on trainee status (eg, year of residency), clinical experience (eg, trainee, advanced practice provider, and attending), and gender. After verbal consent was obtained, usability evaluation was performed virtually using an audio-video platform and the sessions were recorded.

#### Pre-LOOP Survey

Participants first completed a brief anonymous electronic survey. The survey assessed demographics, current method(s) used for patient outcomes review, baseline knowledge of patient outcomes, and attitudes about their current method(s) of review and outcomes (Multimedia Appendix 2). To assess knowledge as opposed to memory, participants were advised prior to the usability evaluation to bring any materials they use to track their patient outcomes and encouraged to refer to these aids to demonstrate their knowledge about their patient outcomes during the survey, which required participants to estimate the frequency of postencounter patient outcomes over several time periods. We assessed their attitudes about patient outcomes by asking about the confidence in estimates, ease of finding this information, and usefulness of knowing this information. Furthermore, we asked about their willingness to use their current method to find these outcomes, their trust in the data obtained, and whether the information collected is representative of the overall trends for all their patients.

#### Task Analysis

We then provided participants access to LOOP and performed task analysis while they used the tool. The first set of tasks was determining the number of patients they had seen over the past 30 days who experienced each outcome of interest. Additionally, we asked users to navigate to the chart of a patient who returned within 72 hours and was admitted within our EHR. We also asked them to identify a patient who had died during their hospitalization and email the patient’s information to a member of our team (to simulate informing a colleague about a patient outcome). Lastly, we asked the users to locate the list of all patients they had seen in the past 30 days and to identify a patient who was dispositioned to hospital observation. While completing these tasks, participants were asked to use the “think-aloud” method, verbalizing their thought process. Research team observers assessed usability performance metrics such as task completion time and methods of navigation and identified struggle points.

#### Post-LOOP Survey and Interview

After using LOOP, participants were asked to complete a second survey to assess their knowledge, skills, and attitudes related to using LOOP. This survey asked the same questions as the initial survey and assessed their experience using LOOP. To assess usability of the tool, we combined and adapted the System Usability Scale [16], the Standardized User Experience Percentile Rank Questionnaire [17], and validated instruments composed of statements, and asked users to indicate their level of agreement: strongly disagree, disagree, agree, strongly agree (Multimedia Appendix 2). In the final step of the usability evaluation, we performed semistructured interviews to debrief with the participant about their experience with LOOP related to the perceived benefits, usefulness, and intention to use (Textbox 1). At the end of each usability evaluation, we asked for feedback about their usability evaluation experience, and observers had the opportunity to ask follow-up questions about observations from the task analyses.

**Questions from a usability evaluation semistructured interview.**

1. Overall, how would you describe your experience with the Linking Outcomes Of Patients (LOOP)?
2. I know you were asked in the surveys about ease—do you find the interface easy to use? Why?
3. Did you find any aspects difficult? What did you expect to happen?
4. How do you feel about the level of information being shown in the outcomes? Do you find the information easy or difficult to digest? Why?
5. Was there any information you found surprising?
6. Do you find LOOP is more or less effective than your current method of reviewing patients? Why? About how long would you estimate you spend on your current method?
7. Are there other benefits you see to using this tool?
8. When do you see yourself using LOOP? What feature or addition that would bring you back to using LOOP?

#### Data Analysis

After each usability evaluation session, the research team debriefed, uploaded their data to a secure team folder, and collectively summarized the performance metrics, key findings, and issues to address by comparing notes to reach consensus. Issues raised by users during testing as well their semistructured interviews were classified as either front-end (design) or back-end (data infrastructure) challenges. Additionally, issues were categorized based on benefit to the user (significant benefit/minimal benefit) and effort to address (easy, intermediate, difficult). For statistical analysis of the preusability and postusability evaluation survey results as well as task times, descriptive statistics were calculated and data were visualized using R 4.0.3 (R Core Team).

## Results

### Phase 1: Conceptual Development Under an HCD Framework

#### Frame and Plan

Through cocreation sessions, the research team came to consensus on a well-defined focus to create a closed feedback loop with postencounter patient-based outcomes for EM providers. The team also determined the steps for accomplishing the remaining stages of the project, which will be further defined below.

#### Research and Synthesis

The design researchers completed 18 person-hours of workflow observations across both ED sites and conducted semistructured interviews of 11 EM clinicians. Attending EM physicians, resident physicians, and advanced practice providers participated in observations and interviews. Surveys were completed by 24 attending EM physicians. Several important themes emerged from observations and interviews, as detailed in Table 2. Many EM clinicians were observed using self-devised work-around solutions to track post-ED encounter patient outcomes, including manual creation of patient lists (electronic or handwritten) to facilitate EHR review in the future and exchange of contact information with patients; others reported similar approaches during interviews. We found that EM clinicians desire information about post-ED encounter patient outcomes and see this type of feedback as important to practice-based learning. They also reported that this information is most often unavailable, and when available, is predominantly negative (ie, associated with adverse patient outcomes). Several clinicians reported that these strategies are only effective when they are time permitted, which is a continuous challenge in the ED environment.

**Table 2:** Themes from observations and semistructured interviews during the research stage of the human-centered design studio.

Theme	Examples of observations	Examples of interview quotations
Emergency Medicine clinicians value postencounter outcomes–driven feedback.	Physician writes down patient’s phone number and reports they do this when they want feedback about that patient’s outcome. Physician gives their personal telephone number to older patients to enable closed-loop feedback.	*…I wish there was a way they could contact me and say, ‘I’m not improving, I’m going to see my primary care provider.’* *…Post encounter feedback is crucial.* (About patient outcome follow-up) *…It’s sort of like a vitamin that I have to take every day for my health. It’s something that will make me a better doctor in the long run.*
Existing systems for delivery of information about patient outcomes are severely limited.	Physician pulls out the list of patients they keep track of from their pocket. Says they are only able to track a couple of patients in each shift.	…*If patients are not put on a list in (the electronic health record) they disappear.* *…The feedback (we receive) is not representative of what is actually happening.*
Currently available outcomes-driven feedback is predominantly negative.	Physician reports that if something bad happens, clinicians find out from leadership. Physician seems tense when discussing.	…*Feedback is limited to lawsuits and bad outcomes.* *…I get feedback if someone complains or dies.*
Emergency Medicine clinicians use workaround solutions to obtain outcome information for cases perceived as interesting or high risk.	Physician leaves patient notes unsigned so that patients’ charts will remain in their electronic health record workflow, forcing additional case review	*…I call patients if I’m concerned about them.* *…I keep a list of patients on my electronic health record profile when I want to see what happened to them.*

These themes were further supported by survey results. The most frequent mode reported for learning about patient outcomes was manual EHR chart review (20 of 24 surveyed EM clinicians), followed by email (7/24), phone (5/24), and face-to-face communications (4/24) between colleagues and patients. A small proportion of EM clinicians (2/24) reported learning about outcomes “haphazardly” and during morbidity and mortality conferences, further reinforcing the idea that most feedback available to EM clinicians is negative. Three-quarters of those surveyed (18/24) preferred to receive both positive and negative feedback, while 6 preferred to receive feedback related to negative outcomes only. Most EM clinicians wanted to know if patients required escalation of care level (eg, transfer from floor to intensive care unit) shortly after admission (20/24), died during their hospitalization (15/24), had a discrepancy between diagnosis assigned at time of admission (ED diagnosis) and diagnosis assigned at hospital discharge (inpatient diagnosis) (14/24) or returned for repeat ED evaluation within a brief time window after ED discharge (16/24). Fewer surveyed clinicians (<30%) wished to be notified when patients filled ED prescriptions, visited an urgent care clinic, followed up with primary care physicians, or had medication treatment regimens changed in the inpatient setting after ED departure.

**Figure 2:**
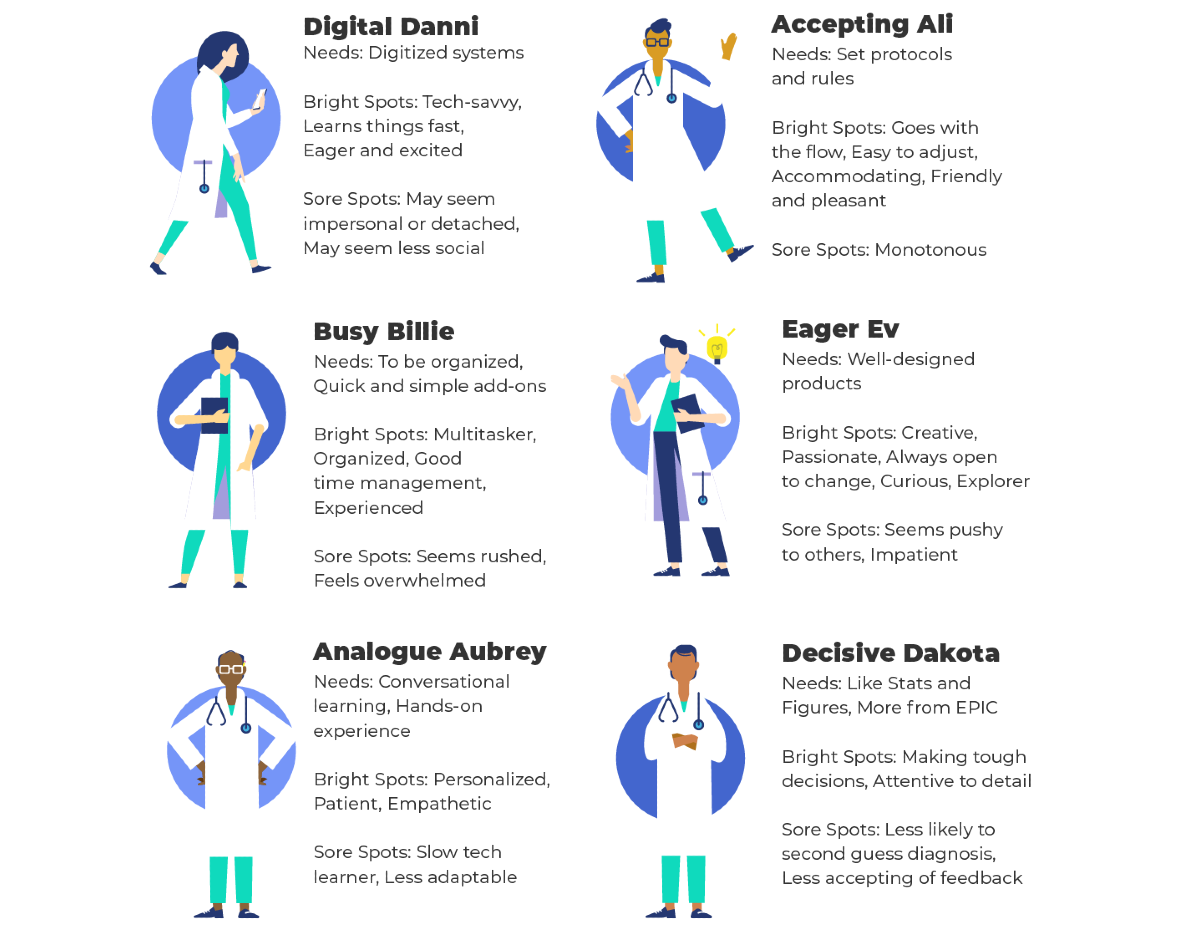
Six user personas generated during human-centered design studios and used to drive tool development and to perform internal prototype testing.

User personas, guiding design principles, and opportunity areas were also defined using information gathered during design research activities. Six user personas that spanned age groups, learning styles, and affinities for technology were generated and used to drive iterative tool design and development and to perform internal testing of prototypes (Figure 2). Design principles around which all future design and development activities would revolve included (1) recognition and demonstration of value for the clinician’s practice, (2) capturing curiosity and encouraging action through knowledge building, (3) prioritization of clear and simple information delivery, and (4) maintenance of flexibility to respond to end user clinicians’ needs and preference as they arise throughout the co-design process. Finally, the 4 primary opportunity areas identified for meaningful impact were (1) provision of balanced positive and negative feedback, (2) provision of feedback in a format that allows for improvement of decision-making without overwhelming clinicians, (3) provision of both population-level and patient-level outcome data, and (4) creation of a platform that is customizable at the individual clinician level.

#### Ideation, Prototyping, and Iteration

**Figure 3:**
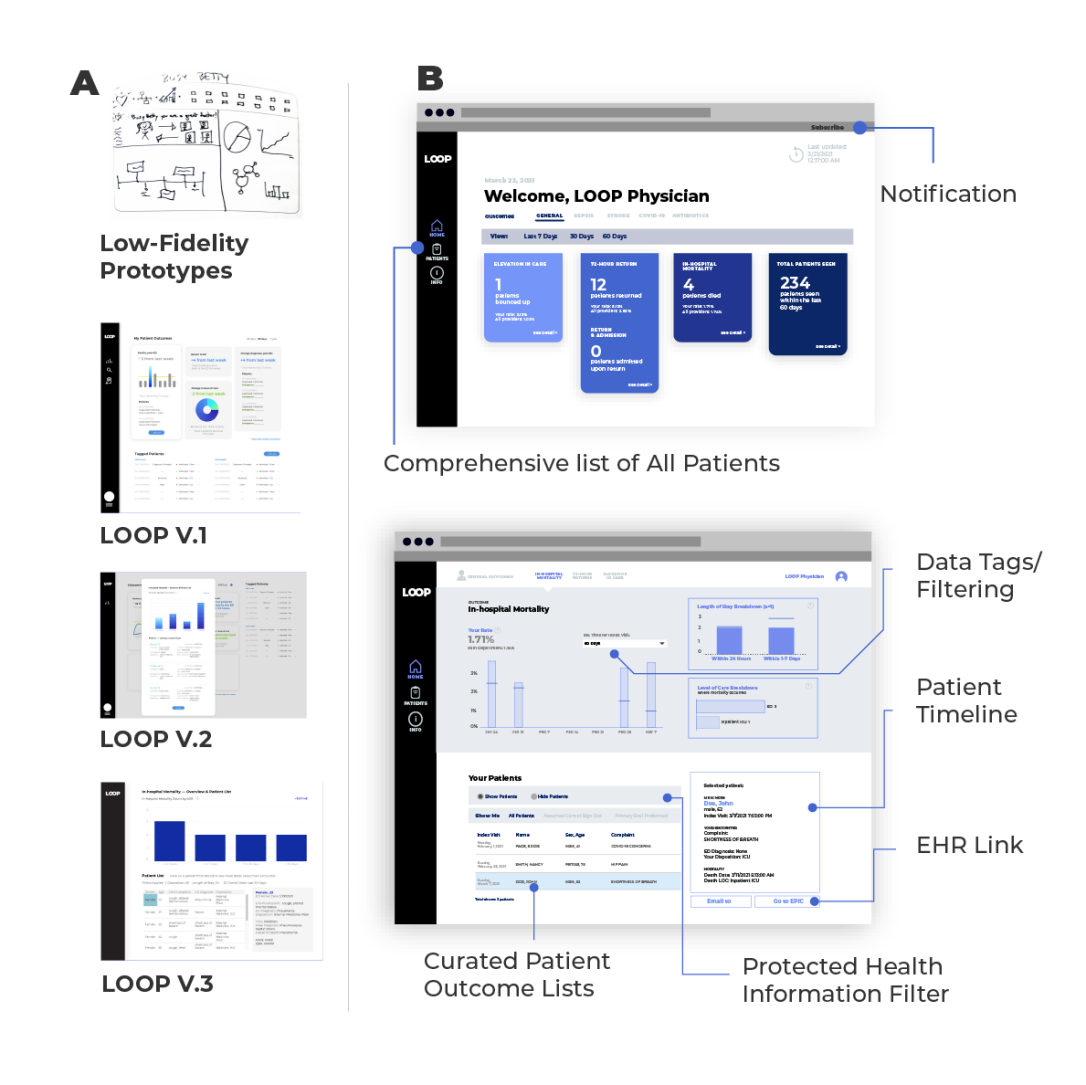
Evolution of Linking Outcomes Of Patients from human-centered design studios going from (A) physician prototypes to the (B) final version used in the usability evaluation. EHR: electronic health record; LOOP: Linking Outcomes Of Patients.

During our multistakeholder ideation session, designers collaborated with engineers, data specialists, and end user EM clinicians to translate the *themes, opportunity areas,* and *design principles* above into a set of target *design features* that would guide analog prototyping of our tool. Design features that emerged from this session are shown in Table 3. Each participating EM clinician was then assigned a clinician *user persona*, and a 2D sketch representation (first prototype) of a feedback platform was generated for that persona (see Figure 3 for a representative example). Although all design features were represented across the user-generated prototypes, only *comprehensive patient lists*, *data tagging,* and *EHR interoperability* were observed in all prototypes. Over several additional weeks, designers analyzed user prototypes and developed a final analog prototype that included as many design features as possible through iteration.

**Table 3:** Design requirements established during ideation and prototyping phases of our human-centered design studio.

Design features	Purpose
Comprehensive lists	Allows users to find information of all the patients they have cared for
Electronic health record interoperability	Allows users to transition between platform and patient’s clinical chart
Data tagging	Gives users the power to drill down through the use of data labels/tags
Pin a patient	Allows users to prioritize patients of interest for future review
Glow moments	Allows other users to appreciate the work of fellow clinicians
Task timer	Allows a user to customize their experience based on time availability
Home/hospital toggle	Limits visibility of protected health information outside of hospital setting
Patient timeline	Allows users to see a patient’s journey after their care
Notification/reminder	Allows users to set an alarm to review outcomes

### Phase 2: Technical Platform Development

As shown in Figure 3, analog prototypes were transitioned to a digital feedback platform in a stepwise fashion. The first digital versions of LOOP were developed using design software that was not connected to real-time data flows. Earliest version (Figure 3A) development focused on incorporation of design features defined during phase 1 ideation, while later versions focused on establishing dimensionality that would facilitate attribution of patients to individual clinicians and allow for data sorting and filtering by time and outcome (Figure 3A). Finally, the digital platform was translated into analytic software used by our health care system (Figure 3A) and optimized to accept real-time feeds of normalized EHR data. End user EM clinicians, engineers, and data analysts were included at every stage of digital development. Engineers and data analysts ensured the platform was technically feasible, while end users ensured it was functional and maintained consistency with the themes and design principles generated during our HCD studio. Most design features that were incorporated into early end user protypes (Table 3) were included in the final version of LOOP, including *comprehensive lists*, *EHR interoperabilit*y, *data tagging,* and *home/hospital toggle* (Figure 3B). Other features, including *pin a patient*, *task timer,* and *notification reminder*, were not included as explicit features of the final platform, but tasks associated with these features were possible to perform within the platform using other mechanisms. Others, including *glow moments* and *patient timeline,* were not included in the final platform owing to technical limitations, but they are features that we will seek to incorporate in future versions.

### Phase 3: Usability Evaluation

For usability evaluation, our study population included 12 EM providers and 6 (50%) were women. There were 3 (25%) attending physicians, 7 (58%) resident physicians, and 2 (17%) advanced practice providers. The median age was 33.5 (IQR 28-38.3) years. Usability evaluation sessions ranged from 30 to 45 minutes in duration.

#### Pre-LOOP Survey

**Figure 4:**
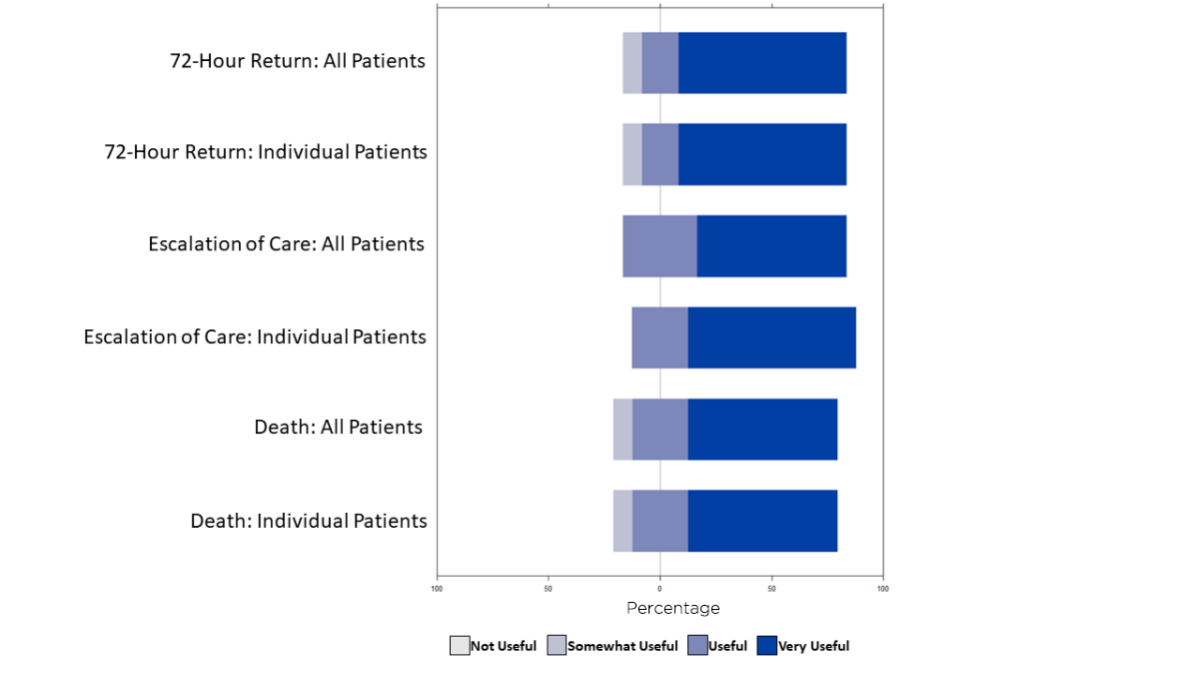
Participant attitudes about usefulness of access to outcomes at individual patient level and across all their patients.

When asked about their current method for identifying outcomes for their patients, the median time spent per week to follow-up on patient outcomes was 1.5 (range 0.5-3.5) hours. Of the 12 EM providers, 9 (75%) that described their current method is manually adding each patient to custom-made lists within the EHR; 2 (17%) stated they make handwritten lists of their patients, and 1 (8%) had no method for tracking patient outcomes. Participant attitudes about their current method for determining the outcomes for their patients indicated there was room for improvement. When asked their level of agreement to the statement “I am likely/willing to review my patients using my current method,” 11 (92%) users either “strongly disagreed” or “disagreed;” 8 (67%) users disagreed with the statement “I trust the data I am able to find on my patients using my current method.” Most users (9/12, 75%) “agreed” or “strongly agreed” that the data gathered using their current method were representative of the overall trends for all their patients. Figure 4 shows participant attitudes about the usefulness of access to outcomes. Most users reported that being able to access all 3 outcomes was “very useful” at the individual patient level and for all their patients.

#### Task Analysis

The task analysis of the participants using LOOP to find all 3 outcomes for their patients from the last 30 days was completed in a median of 1.09 (range 0.7-4.3) minutes. The median time to complete all 3 special function tasks (eg, navigating to the electronic record from LOOP, emailing patient information to a colleague, navigating filter features of LOOP) was 6.9 (range 1.7-12.2) minutes. Examples of important observations during this task analysis were (1) the user spent time interpreting a graph instead of noticing the summarizing number somewhere else on the screen and (2) data display errors.

#### Post-LOOP Survey and Interview

**Figure 5:**
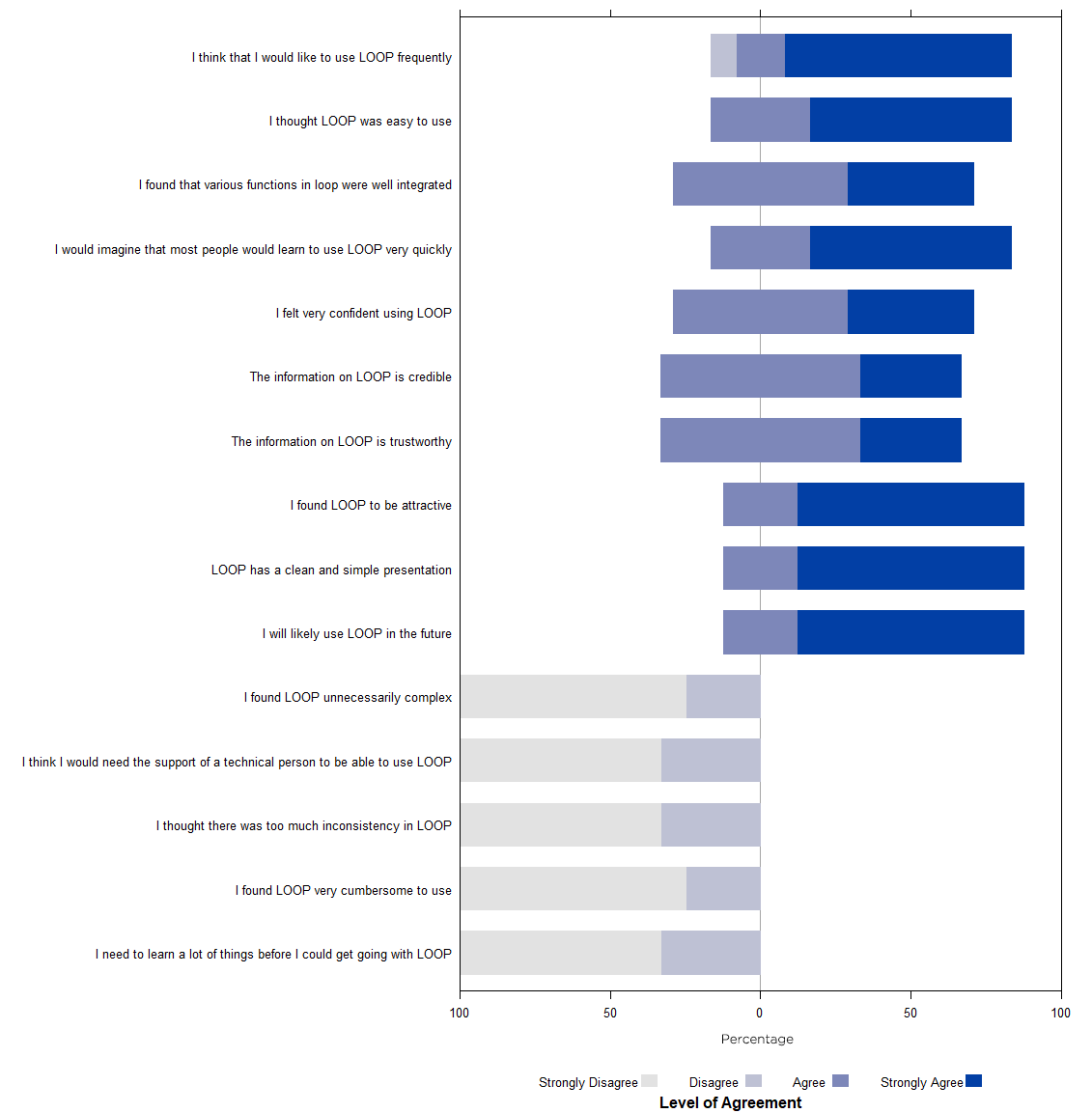
Participant attitudes about Linking Outcomes Of Patients. LOOP: Linking Outcomes Of Patients.

Post-LOOP survey completion time was a median of 1.8 (range 1.1-2.7) minutes. Participants’ knowledge of the number of patients that experienced each outcome was different from observed outcomes in LOOP (Figure S1 in Multimedia Appendix 3). Participants underestimated the number of patients who died in hospital but overestimated the number who required an escalation of care outcome. Of note, 1 participant was excluded owing to nonresponse on the initial survey (Figure S2 in Multimedia Appendix 4). The participants’ attitudes about their estimates of each patient outcome over the past 30 days improved after using LOOP (Figure S2 in Multimedia Appendix 4). Of note, 1 participant was removed from the analysis for only the question “How easy is it for you to determine this outcome for all your patients?” for all 3 outcomes owing to nonresponse on the post-LOOP survey. For all 3 outcomes, the users shifted from feeling the outcomes were “not easy” to determine at the individual and cumulative patient levels to feeling it was “very easy” after using LOOP. Additionally, they changed from feeling “not confident” and “somewhat confident” about their estimates to overall “very confident” after using LOOP. Participant attitudes about LOOP were overall favorable (Figure 5). On a scale from 0 (unlikely) to 10 (most likely), the users were likely to recommend (score=9.5) LOOP to a colleague. The semistructured interview to debrief with the users about their LOOP experience helped further inform our understanding of their perceptions about LOOP. The users identified several benefits about LOOP, such as access to data on patients you would not have originally had the time or foresight to follow up on later. One user described LOOP as “much more systematic” than their current methods. Importantly, trainees identified the opportunity to use LOOP as an educational tool that facilitates discussion with their attendings about prior cases with surprising outcomes. A user commented that LOOP is a “fantastic learning tool to make you a better clinician.” Regarding the issues identified, the constructive feedback received was helpful and could be addressed. Many concerns centered around harmonizing the visual layout with the functionality on the main page as well as across the platform (eg, connecting summary numbers with the corresponding patient lists). Another issue was improving the defaults and layouts of filtered lists; therefore, the interaction was more intuitive. The ability to discuss with the users while having the tool in front of them to demonstrate the concerns and possible improvements was highly informative.

## Discussion

### Principal Results

Leveraging previously underutilized EHR data, LOOP was designed and developed to enable systematic delivery of personalized patient outcomes feedback to EM clinicians. The platform allows EM clinicians to (1) quickly review post-ED encounter outcomes at the individual patient level, (2) see outcomes for all patients in their census, and (3) customize views of data based on user preference. Our usability evaluation showed the tool is easy to use and that information presented within LOOP is viewed as valuable and reliable by end users. The usability evaluation also revealed that information delivered within LOOP is not currently known by EM clinicians. Our team’s creation of a tool that is both useful and usable was enabled by a process centered on HCD principles and by a commitment to incorporation of end users at every stage of design and development. Although the version of LOOP reported here includes a selected set of outcomes only (in-hospital mortality, inpatient level of care escalations, and return ED visits), the tool was designed with flexibility to allow for ongoing rapid integration of additional outcomes based on user feedback and clinical need.

### Comparison With Prior Work

Absence of patient outcomes feedback in EM is well-documented as is recognition of the importance of this information and a desire to receive it among EM clinicians [6,10,17,26]. Detailed information related to patient outcomes is routinely collected and stored within the EHR data infrastructure; opportunity exists to enable practice-based learning and improvement through standardization and delivery of this information to clinicians [19,20]. However, most efforts to provide patient outcomes feedback in EM have relied on analog and nonsystematic approaches such as manual creation of patient follow-up lists or telephone calls to patients by clinicians [18,27-29]. These approaches are labor-intensive and time-consuming. They also have the potential to promote cognitive bias. When tasked with selecting patients for follow-up themselves, clinicians often focus on cases they already perceived as challenging or at the highest risk for adverse outcome [30,31]. The automated and comprehensive method of data delivery used by LOOP ensures that clinicians receive robust unbiased outcome data, including information that is unexpected and not discoverable using previously described methods. Exposure of these outcomes is important for both early career and seasoned clinicians because unexpected events uncover unconscious deficits and present opportunities to improve competence and increase the quality and safety of care delivered [18,32-34].

Prior efforts to harness EHR data for EM practice improvement have focused on the development of dashboards to guide ED operations management or to enhance real-time display of patient data during the ED encounter [35-37]. To our knowledge, LOOP is the first tool that translates EHR data into post-ED encounter patient outcomes feedback for individual EM clinicians. The systematic delivery of these data by LOOP facilitates deliberate practice in EM, a process whereby expertise can be developed through repeated action and skills improvement, driven by continual feedback and reassessment [7,38,39]. Such practice-based learning is critical to the personal and professional development of clinicians and is mandated by both the ACGME and ABMS [2,3]. The generation and automation of personalized EHR data flows to fill outcome knowledge gaps is a significant step forward for experiential learning in EM. It also represents a step toward more meaningful use of the EHR and development of a learning health care system, which are both the major goals for our nation’s health care system [40].

### Pragmatic Usability Evaluation

While the potential value of information delivered by LOOP is clear, its real-world utility is dependent on end-user acceptance and long-term adoption. User interface and information display greatly impact whether a tool is adopted by the user [41]. Activities associated with our HCD studio revealed high variation among potential users of LOOP (EM clinicians) with respect to clinical experience, experience and comfort with technology, current practice-based learning behaviors, and desired patient outcomes feedback—all of which were considered during our HCD process. We used a robust and pragmatic approach to assessment that allowed for evaluation of LOOP in a near real-world setting. Our assessment, grounded by the knowledge, skills, and attitudes framework [42], included direct observation and task analysis as users interacted with the tool to find information about real patient encounters, surveys that included standard usability questions and assessed knowledge and attitudes that allowed for comparison with current practices, and semistructured interviews that further explored these topics. We also performed this assessment among a diverse and representative group of end users. Our findings were almost exclusively positive.

### Human-Centered Design (HCD)

Our commitment to the use of HCD methods at every stage of this project was critical to its success. The incorporation of user input into the development of information technology platforms is now considered essential in health care [43]. Previously reported clinician user involvement in similar projects is variable, with some groups limiting their involvement to ideation, implementation, or testing phases only [36,37,44]. To our knowledge, the HCD methodology used here is among the most intentional and extensive of those reported to date. This approach was enabled by our multidisciplinary team structure. Longitudinal collaboration between clinician-scientists, engineers, and designers allowed for interpretation of end user contributions through multiple lenses and ensured that the final product of our work was a well-rounded representation of user-generated specifications. We believe this increased end user trust in the final product and will translate to higher rates of adoption in clinical practice. The results of our usability evaluation suggest this is true.

### Limitations

This study has several limitations. First, the study was performed within a single health care system, which may limit its generalizability. However, our incorporation of end user EM clinicians throughout design, development, and evaluation activities from varied practice settings (urban academic and suburban community) and levels of training (attending EM physicians, resident physicians, advanced practice providers) minimizes this limitation. In addition, our HCD focus and technical approach to EHR data normalization and presentation are both reproducible and generalizable. Feedback platforms that integrate the needs and wants of clinical staff could be generated by other groups using a similar methodological approach. Second, our usability evaluation was performed in a relatively small sample of EM clinicians. This limitation was minimized through inclusion of a diverse and representative user group and by inclusion of various qualitative assessment techniques, which included surveys, direct observations, and semistructured interviews. Our sample size was consistent with those previously reported by others and sufficient to reach thematic saturation using these methods [45,46]. Finally, this study did not evaluate long-term adoption rates or the effectiveness of LOOP for clinical practice improvement. These are both important ongoing research objectives of our team. LOOP is currently in use across multiple EDs, and data collection to facilitate study of these questions is underway.

### Conclusions

This study demonstrates the potential of HCD in EM and the power of EHR data to augment practice-based learning in episodic care environments. We have outlined a framework for working alongside end-user EM clinicians to develop and test a tool that augments their clinical experience and exposes previously unavailable information to create a closed-loop feedback-driven learning platform. Future objectives include incorporation of additional patient outcomes into LOOP, measurement of long-term adoption rates, and impacts of patient outcomes feedback provided by LOOP on clinical practice.

## Appendix

Multimedia Appendix 1 Survey deployed as part of the human-centered design studio. The survey helped provide more specific data around what outcomes physicians wanted after the encounter for admitted and discharged patients.

Multimedia Appendix 2 Usability evaluation surveys collecting demographic information, assessing current practice, and capturing pre– and post–Linking Outcomes Of Patients perceptions about their patient outcomes. The post–Linking Outcomes Of Patients survey also specifically addressed their experience with Linking Outcomes Of Patients.

Multimedia Appendix 3 Participant knowledge about patient outcomes such as (A) in-hospital mortality, (B) escalation of level of care, and (C) return to the emergency department. There were 12 participants, but one is not applicable owing to no response.

Multimedia Appendix 4 Participant attitudes about their estimates of pre–Linking Outcomes Of Patients and post–Linking Outcomes Of Patients for patients with each outcome. Three questions were asked for each outcome in the presurvey and postsurvey: 1. How easy is it for you to determine for all your patients? 2. How easy is it for you to determine for an individual patient? 3. How confident are you?

## Abbreviations

ABMS: American Board of Medical Specialties
ACGME: Accreditation Council for Graduate Medical Education
CDEM: Center for Data Science in Emergency Medicine
ED: emergency department
EHR: electronic health record
EM: emergency medicine
HCD: human-centered design
LOOP: Linking Outcomes Of Patients
MICA: Maryland Institute College of Art
