# Supplementary material for: A Patient Outcomes–Driven Feedback Platform for Emergency Medicine Clinicians: Human-Centered Design and Usability Evaluation of Linking Outcomes Of Patients (LOOP)
Source: JMIR Hum Factors. 2022 Mar 23;9(1):e30130. doi: 10.2196/30130 (PMC8987968; doi:10.2196/30130)
Supplement: Multimedia Appendix 1 [file humanfactors_v9i1e30130_app1.pdf]

Mark your feelings for us!

HELLO, HOW ARE YOU TODAY?

01. JUST FOR OUR INFORMATION,

GENDER:

YEARS OF EXPERIENCE:

NAME OF HOSPITAL: JHH HCGH

CIRCLE ROLE: Attending Resident PA RN

02. HOW DO YOU CURRENTLY FIND OUT WHAT HAPPENED TO YOUR PATIENTS AFTER THEIR ED VISIT?

(Circle all that apply)

E-MAIL

EHR

PHONE CALL

ONLINE / APP: ____________

FACE TO FACE CONVERSATION

OTHERS: ____________

03. HOW WOULD YOU PREFER TO LEARN WHAT HAPPENED TO YOUR PATIENTS AFTER THEIR ED VISIT?

(Circle all that apply)

WHAT FORMAT?

WHAT KIND OF FEEDBACK?

GOOD BAD

FROM WHOM?

SUPERVISOR PEER DOCTORS NURSES

PATIENTS OTHERS: ____________________

05. WHAT POST-ED VISIT INFORMATION ABOUT DISCHARGED PATIENTS WOULD HELP YOU MAKE BETTER DECISIONS FOR FUTURE PATIENTS?

(Choose top five information. If you choose the information contains a blank space, please fill the blank)

- [ ] Deaths within _____ days of ED departure?
- [ ] Patients who filled prescriptions for antibiotics?
- [ ] Patients who visited a primary care doctor within ______ days?
- [ ] Patients who visited an urgent care center within ______ days?
- [ ] Patients who returned to any ED within _____ hours?
- [ ] Patients who returned to any ED and were admitted within _____ days?
- [ ] ____

06. WHAT POST-ED VISIT INFORMATION ABOUT ADMITTED / OBSERVED PATIENTS WOULD HELP YOU MAKE BETTER DECISIONS FOR FUTURE PATIENTS?

(Choose top five information. If you choose the information contains a blank space, please fill the blank)

- [ ] Deaths within _____ days of ED departure?
- [ ] In-hospital death?
- [ ] Initiation of NIPPV or intubation and mechanical ventilation?
- [ ] New initiation or discontinuation of antibiotics?
- [ ] Change in antibiotics (spectrum)?
- [ ] Change in antibiotics (dose)?
- [ ] Change in level of care within _____ hours of admission?
- [ ] Conversion from observation to admission status?
- [ ] Admitted patients who do not meet payer admission criteria?
- [ ] Discrepancy between admitting ED diagnoses and hospital discharge diagnoses?
- [ ] Patients who develop Sepsis in-hospital?
- [ ] ____

07. WHAT THINGS (INDIVIDUAL, INSTITUTIONAL) CURRENTLY COMPLICATE YOUR DECISION MAKING?

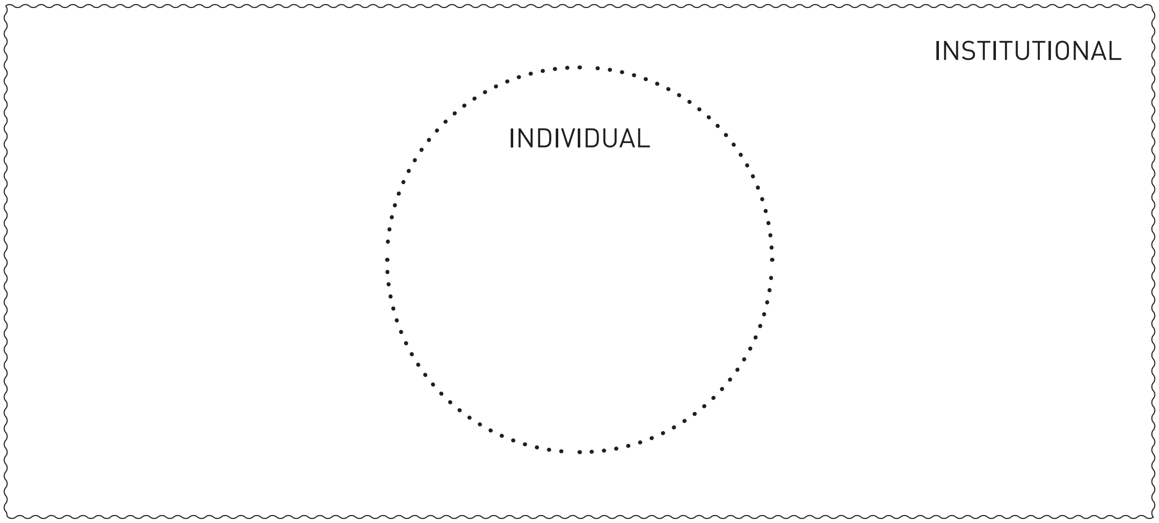

"THANK YOU FOR PARTICIPATING IN THIS SURVEY!"
