# Supplementary material for: A Patient Outcomes–Driven Feedback Platform for Emergency Medicine Clinicians: Human-Centered Design and Usability Evaluation of Linking Outcomes Of Patients (LOOP)
Source: JMIR Hum Factors. 2022 Mar 23;9(1):e30130. doi: 10.2196/30130 (PMC8987968; doi:10.2196/30130)
Supplement: Multimedia Appendix 2 [file humanfactors_v9i1e30130_app2.docx]

Initial Survey

Start of Block: Demographics

Q1 What PGY are you?

- PGY1 resident physician (1)
- PGY2 resident physician (2)
- PGY3 resident physician (3)
- PGY4 resident physician (4)
- Attending physician 5 or less years post-residency (5)
- Attending physician >5 years post-residency (6)
- APP 5 or less years post-training (7)
- APP >5 years post-training (8)

Q3 How old are you? (number only)

________________________________________________________________

Q4 What gender do you identify as?

- Man (1)
- Woman (2)
- Non-Binary (3)
- Prefer not to answer (4)

End of Block: Demographics

Start of Block: Knowledge

Q12 What is your current method for identifying outcomes of your patients related to whether they died in the hospital, had an escalation of care, or returned to the ED w/in 72 hours? (Multi-select)

- Hand-written lists (1)
- EPIC custom made lists that I add patients to (2)
- Make list outside of EPIC but computerized (email, word, etc) (3)
- I don't have a method to find these outcomes for any of my patients (4)
- Other. Please describe in as much detail as possible and/or elaborate on above choices. (5) ________________________________________________

Q13 How many hours do you spend per week following up on your patient outcomes (i.e., not for charting purposes)?

________________________________________________________________

Q5 Click to write the question text

|  | Of the patients you've seen in the last 30 days, what is your best estimate of how many patients had the following post-ED encounter outcomes? (1) |
| --- | --- |
| Outcome: Died in the hospital (1) |  |
| Outcome: Escalation of level of care (e.g. floor to IMC) within 24h of ED departure (2) |  |
| Outcome: Returned to the ED w/in 72 hours (3) |  |

| Page Break |  |
| --- | --- |

End of Block: Knowledge

Start of Block: Attitude

Q6 How **confident** are you about your estimate of the outcomes?

|  | Not confident (1) | Somewhat confident (2) | Confident (3) | Very confident (4) |
| --- | --- | --- | --- | --- |
| Died in the hospital (1) |  |  |  |  |
| Escalation of level of care (2) |  |  |  |  |
| Returned to the ED w/in 72 hours (3) |  |  |  |  |

Q7
 How **easy** is it for you to determine whether an **individual patient** has experienced these outcomes after receiving care from you in the ED?

|  | Not easy (1) | Somewhat easy (2) | Easy (4) | Very easy (5) |
| --- | --- | --- | --- | --- |
| Died in the hospital (1) |  |  |  |  |
| Escalation of level of care (2) |  |  |  |  |
| Returned to the ED w/in 72 hours (3) |  |  |  |  |

Q9 How **easy** is it for you to identify **every patient** who has experienced these outcomes after receiving care from you in the ED?

|  | Not easy (1) | Somewhat easy (2) | Easy (4) | Very easy (5) |
| --- | --- | --- | --- | --- |
| Died in the hospital (1) |  |  |  |  |
| Escalation of level of care (2) |  |  |  |  |
| Returned to the ED w/in 72 hours (3) |  |  |  |  |

Q8 How **useful**is it for you to be able to access these outcomes for an **individual** patient of interest seen in the last 30 days?

|  | Not useful (1) | Somewhat useful (2) | Useful (3) | Very useful (4) |
| --- | --- | --- | --- | --- |
| Died in the hospital (1) |  |  |  |  |
| Escalation of level of care (2) |  |  |  |  |
| Returned to the ED w/in 72 hours (3) |  |  |  |  |

Q10 How **useful**is it for you to be able to access these outcomes for **all** of your patients seen in the last 30 days?

|  | Not useful (1) | Somewhat useful (2) | Useful (3) | Very useful (4) |
| --- | --- | --- | --- | --- |
| Died in the hospital (1) |  |  |  |  |
| Escalation of level of care (2) |  |  |  |  |
| Returned to the ED w/in 72 hours (3) |  |  |  |  |

Q12 What is your level of agreement with the following statements about your current method to identify these outcomes on your patients?

|  | Strongly disagree (1) | Disagree (2) | Agree (4) | Strongly agree (5) |
| --- | --- | --- | --- | --- |
| "I am likely/willing to review my patients using my current method." (1) |  |  |  |  |
| "I trust the data I am able to find on my patients using my current method." (2) |  |  |  |  |
| "The data I gather using my current method is representative of the overall trends for all my patients for these outcomes and time periods." (3) |  |  |  |  |

End of Block: Attitude

Post-LOOP Survey

Start of Block: Knowledge

Q5 Click to write the question text

|  | After using LOOP, of the patients you've seen in the last 30 days, what is your best estimate of how many patients had the following post-ED encounter outcomes? (1) |
| --- | --- |
| Outcome: Died in the hospital (1) |  |
| Outcome: Escalation of level of care (2) |  |
| Outcome: Returned to the ED w/in 72 hours (3) |  |

| Page Break |  |
| --- | --- |

End of Block: Knowledge

Start of Block: Attitude

Q6 After using LOOP, how **confident**are you about your estimate of the outcomes?

|  | Not confident (1) | Somewhat confident (2) | Confident (3) | Very confident (4) |
| --- | --- | --- | --- | --- |
| Died in the hospital (1) |  |  |  |  |
| Escalation of level of care (2) |  |  |  |  |
| Returned to the ED w/in 72 hours (3) |  |  |  |  |

Q7 Using LOOP, how **easy** is it for you to determine whether an **individual patient** has experienced these outcomes after receiving care from you in the ED?

|  | Not easy (1) | Somewhat easy (2) | Easy (4) | Very easy (5) |
| --- | --- | --- | --- | --- |
| Died in the hospital (1) |  |  |  |  |
| Escalation of level of care (2) |  |  |  |  |
| Returned to the ED w/in 72 hours (3) |  |  |  |  |

Q11 Using LOOP, how **easy** is it for you to identify **every patient** who has experienced these outcomes after receiving care from you in the ED?

|  | Not easy (1) | Somewhat easy (2) | Easy (4) | Very easy (5) |
| --- | --- | --- | --- | --- |
| Died in the hospital (1) |  |  |  |  |
| Escalation of level of care (2) |  |  |  |  |
| Returned to the ED w/in 72 hours (3) |  |  |  |  |

Q9 Using LOOP, what is your level of agreement with the following statements about the method you just used to identify these outcomes on your patients?

|  | Strongly disagree (1) | Disagree (2) | Agree (4) | Strongly agree (5) |
| --- | --- | --- | --- | --- |
| "I think that I would like to use LOOP frequently." (1) |  |  |  |  |
| "I found LOOP unnecessarily complex." (2) |  |  |  |  |
| "I thought LOOP was easy to use." (3) |  |  |  |  |
| "I think that I would need the support of a technical person to be able to use LOOP." (5) |  |  |  |  |
| "I found that various functions in LOOP were well integrated." (6) |  |  |  |  |
| "I thought there was too much inconsistency in LOOP." (7) |  |  |  |  |
| "I would imagine that most people would learn to use LOOP very quickly." (8) |  |  |  |  |
| "I found LOOP very cumbersome to use." (9) |  |  |  |  |
| "I felt very confident using LOOP." (10) |  |  |  |  |
| "I needed to learn a lot of things before I could get going with LOOP." (11) |  |  |  |  |
| "The information on LOOP is credible." (12) |  |  |  |  |
| "The information on LOOP is trustworthy." (13) |  |  |  |  |
| "I found LOOP to be attractive." (14) |  |  |  |  |
| "LOOP has a clean and simple presentation." (15) |  |  |  |  |
| "I will likely use LOOP in the future." (16) |  |  |  |  |

Q12 How likely are you to recommend LOOP to a friend or colleague? (0=not at all likely; 10=extremely likely)

|  | 0 | 1 | 2 | 3 | 4 | 5 | 6 | 7 | 8 | 9 | 10 |
| --- | --- | --- | --- | --- | --- | --- | --- | --- | --- | --- | --- |

| 1 () | 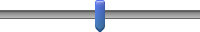 |
| --- | --- |

End of Block: Attitude
