# Supplementary figures and images for: A Patient Outcomes–Driven Feedback Platform for Emergency Medicine Clinicians: Human-Centered Design and Usability Evaluation of Linking Outcomes Of Patients (LOOP)
Source: JMIR Hum Factors. 2022 Mar 23;9(1):e30130. doi: 10.2196/30130 (PMC8987968; doi:10.2196/30130)

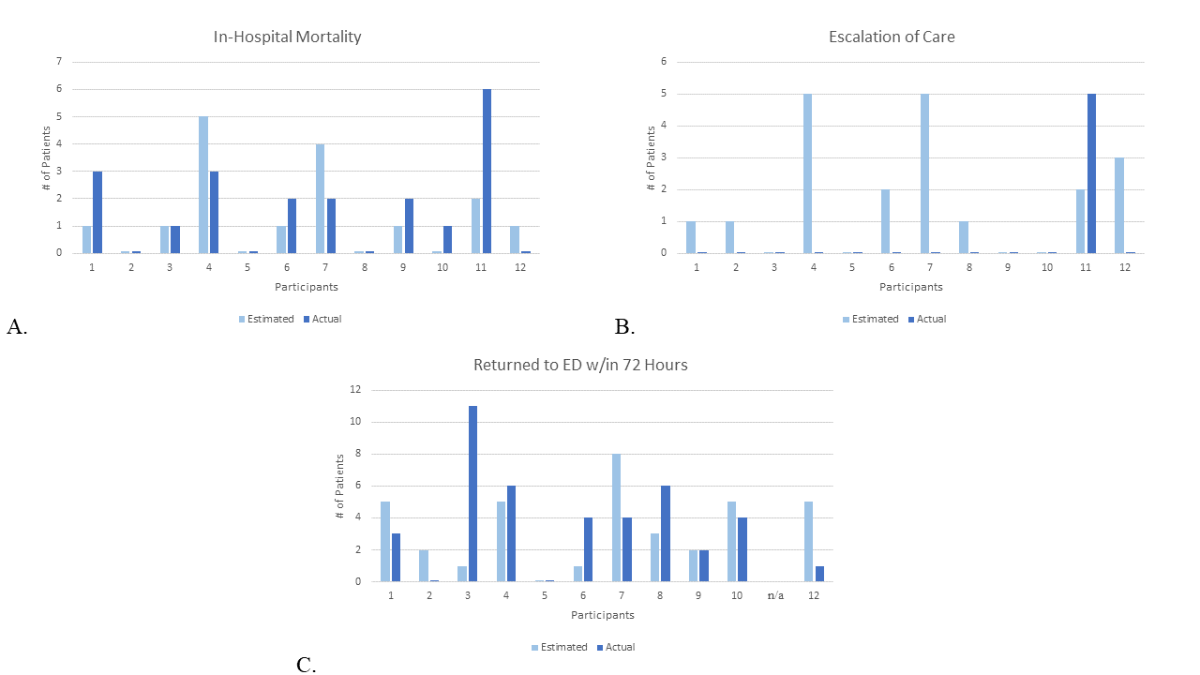

Supplement: Multimedia Appendix 3 [file humanfactors_v9i1e30130_app3.png]

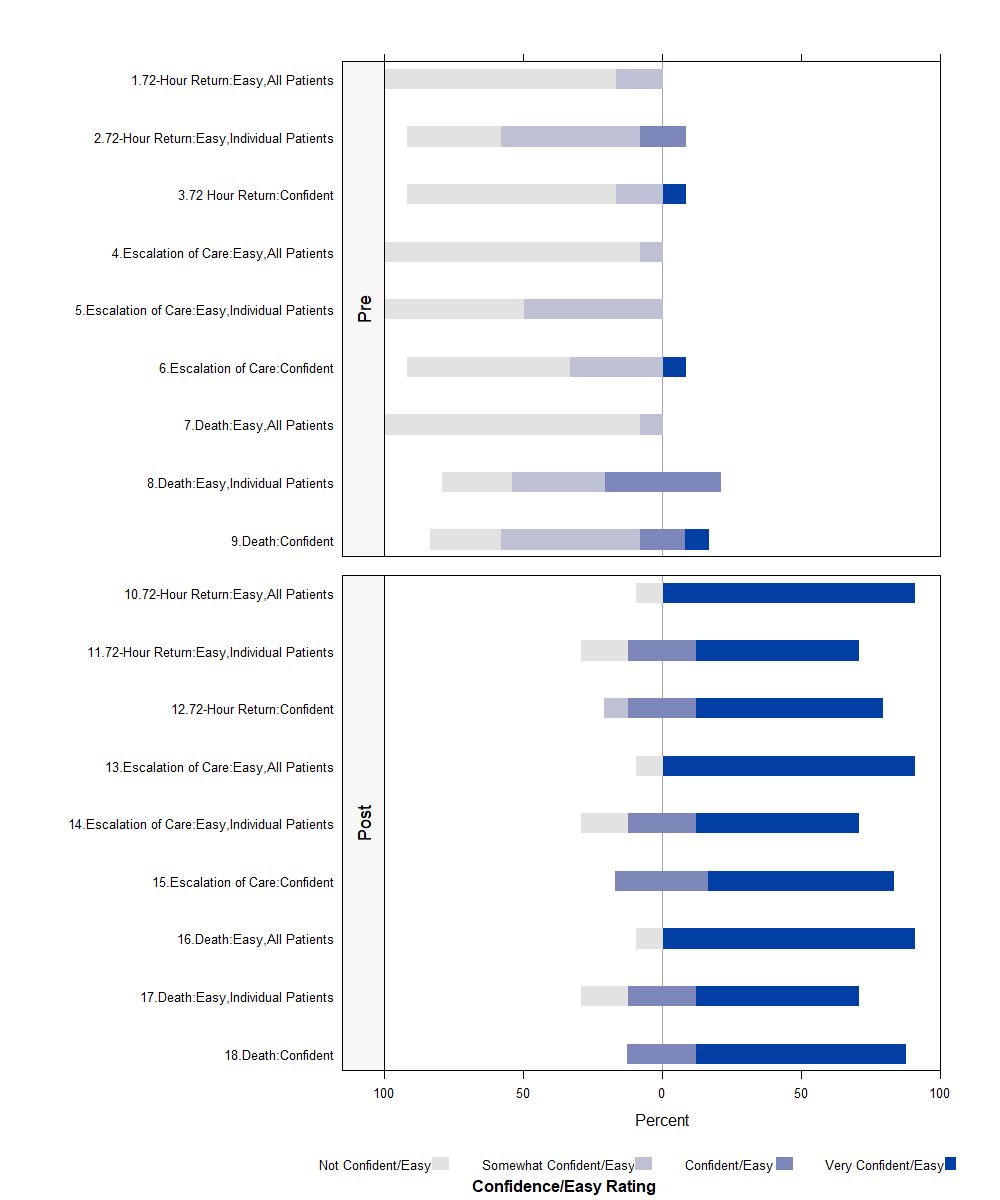

Supplement: Multimedia Appendix 4 [file humanfactors_v9i1e30130_app4.png]
